# Supplementary material for: Realization of High Current Gain for Van der Waals MoS2/WSe2/MoS2 Bipolar Junction Transistor
Source: Nanomaterials (Basel). 2024 Apr 19;14(8):718. doi: 10.3390/nano14080718 (PMC11053443; doi:10.3390/nano14080718)
Supplement: Supplementary file 1 [file nanomaterials-14-00718-s001.zip › nanomaterials-2936866-supplementary.pdf]

nanomaterials

Supplementary Material

Realization of High Current Gain for Van der Waals $MoS_2/WSe_2/MoS_2$ Bipolar Junction Transistor

Zezhang Yan, Ningsheng Xu and Shaozhi Deng *

State Key Laboratory of Optoelectronic Materials and Technologies, Guangdong Province Key Laboratory of Display Material and Technology, School of Electronics and Information Technology, Sun Yat-sen University, Guangzhou 510275, China
* Correspondence: stsdsz@mail.sysu.edu.cn (S.D.)

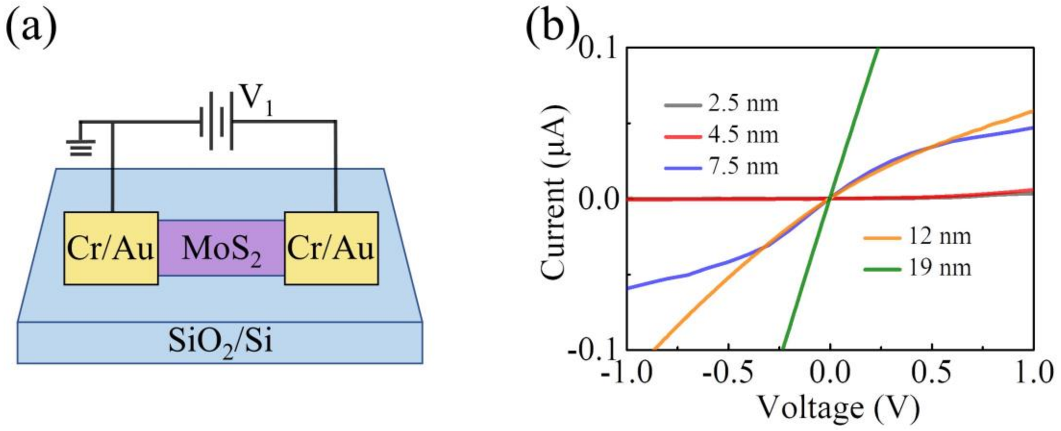

Figure S1. (a) A schematic illustration of the electrical connection to investigate the electrical characteristics of the undoped MoS_2 flakes with different thicknesses. (b) Typical I–V curves of the undoped MoS_2 flakes with different thicknesses.

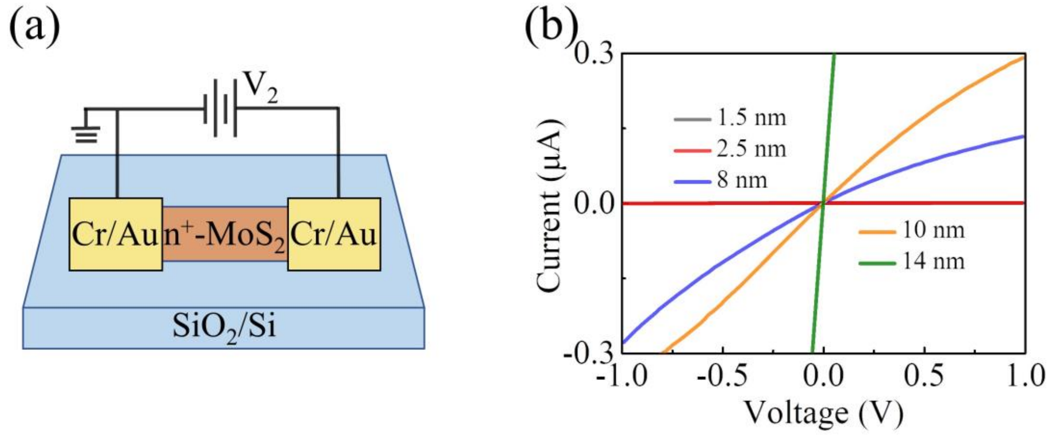

Figure S2. (a) A schematic illustration of the electrical connection to investigate the electrical characteristics of the re-doped MoS_2 flakes with different thicknesses. (b) Typical I–V curves of the re-doped MoS_2 flakes with different thicknesses.

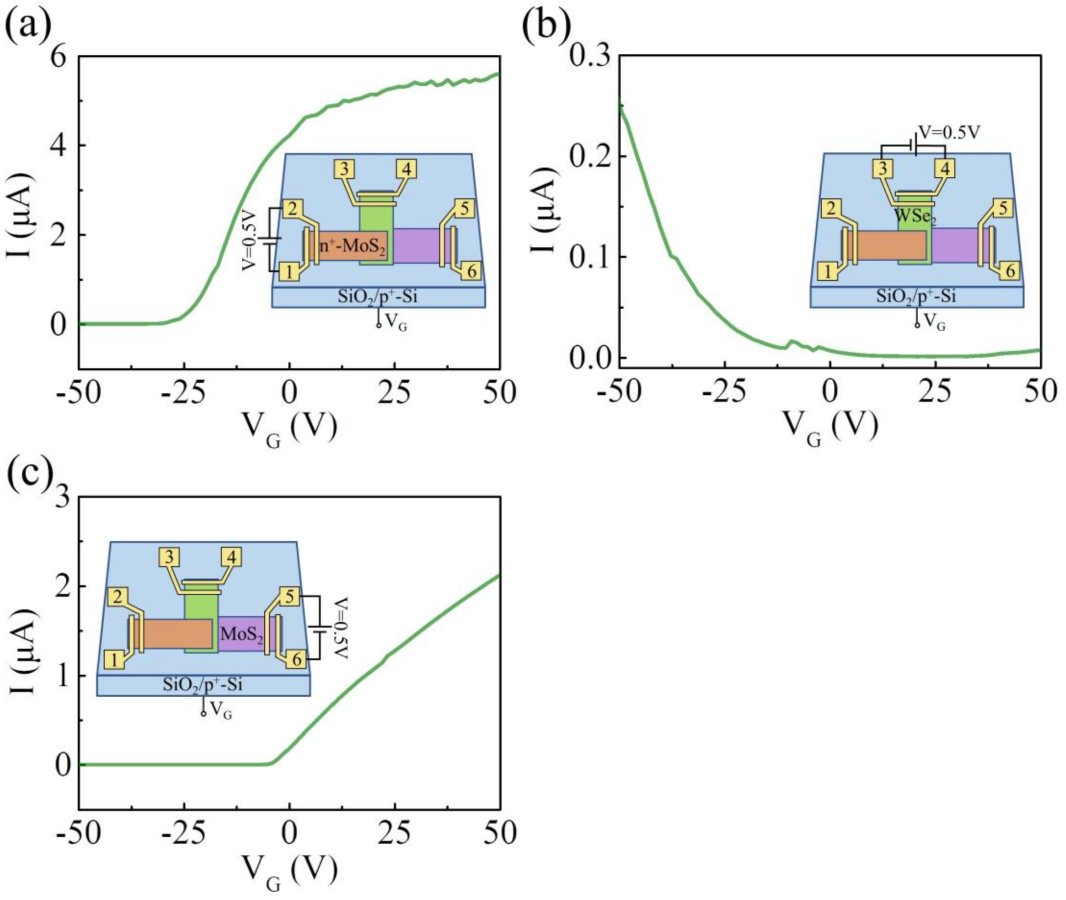

Figure S3. (a) The transfer curve of the individual n^+-MoS_2 flake; (b) The transfer curve of the individual WSe_2 flake; (c) The transfer curve of the individual MoS_2 flake.

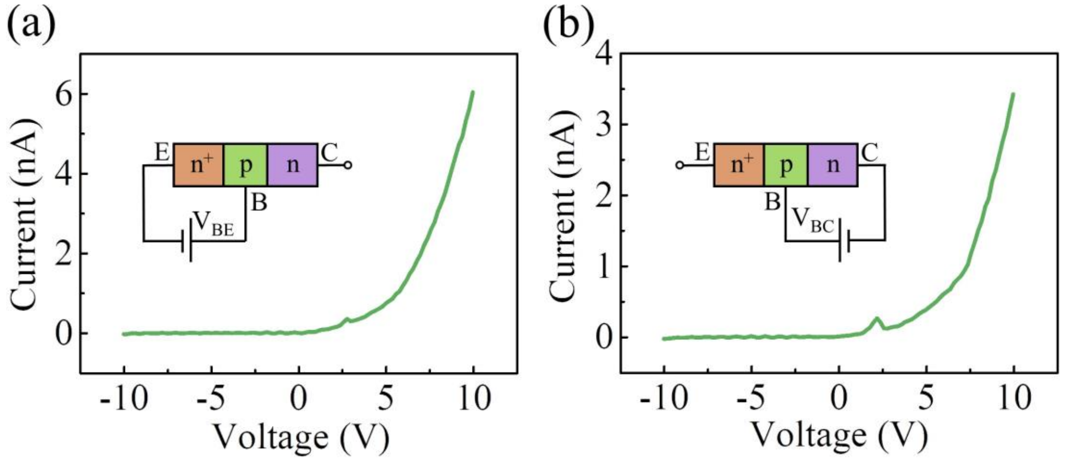

Figure S4. (a) I–V curves of the base-emitter (top-n^+-MoS_2/WSe_2) junction; (b) I–V curves of the base-collector (bottom-MoS_2/WSe_2) junction.

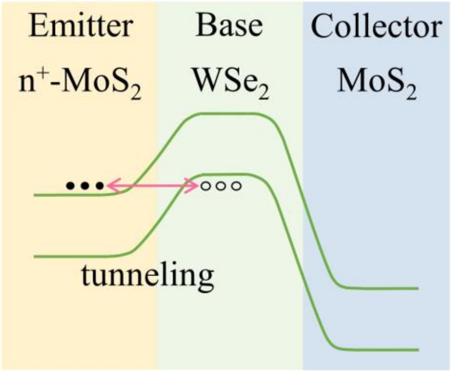

Figure S5. Band diagram of the n^+-MoS_2/WSe_2/MoS_2 bipolar junction transistor operating in the negative differential resistance region

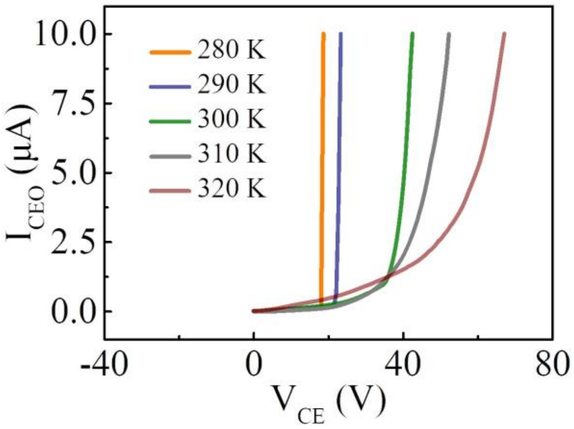

Figure S6. The open-base collector-emitter breakdown characteristics of the device operating at different temperatures (from 280 K to 320 K).
